# Oropharyngeal Muscle Exercise Therapy Improves Signs and Symptoms of Post-stroke Moderate Obstructive Sleep Apnea Syndrome

**DOI:** 10.3389/fneur.2018.00912

**Published:** 2018-10-29

**Authors:** Dongmei Ye, Chen Chen, Dongdong Song, Mei Shen, Hongwei Liu, Surui Zhang, Hong Zhang, Jingya Li, Wenfei Yu, Qiwen Wang

**Affiliations:** ^1^Department of Rehabilitation, Affiliated Zhongshan Hospital of Dalian University, Dalian, China; ^2^Department of Anatomy, Medical College of Dalian University, Dalian, China; ^3^Department of Imaging, Affiliated Zhongshan Hospital of Dalian University, Dalian, China; ^4^Department of Rehabilitation, People's Hospital of Longhua District of Shenzhen, Shenzhen, China

**Keywords:** MRI, obstructive sleep apnea syndrome, oropharyngeal muscles exercise, polysomnography, stroke

## Abstract

The primary aim of the current study was to assess the effects of oropharingeal muscle exercises in obstruction severity on stroke patients with OSAS. The secondary aims were to evaluate the effects of the exercises on rehabilitation of neurological function, sleeping, and morphology change of upper airway. An open-label, single-blind, parallel-group, randomized, controlled trial was designed. Fifty post-stroke patients with moderate OSAS were randomly assigned into 2 groups (25 in each group). For the therapy group, oropharyngeal muscle exercise was performed during the daytime for 20 min, twice a day, for 6 weeks. The control group was subjected to sham therapy of deep breathing. Primary outcomes were the obstruction severity by polysomnography. Secondary outcomes included recovery of motor and neurocognitive function, personal activities of daily living assessment (ADL), sleep quality and sleepiness scale. It also included upper airway magnetic resonance imaging (MRI) measurements. Assessments were made at baseline and after 6-week exercise. Finally, 49 patients completed the study. The apnea–hypopnea index, snore index, arousal index, and minimum oxygen saturation improved after exercise (*P* < 0.05). Oropharyngeal muscle exercises improved subjective measurements of sleep quality (*P* = 0.017), daily sleepiness (*P* = 0.005), and performance (both *P* < 0.05) except for neurocognition (*P* = 0.741). The changes in obstruction improvement, sleep characteristics and performance scale were also associated with training time, as detected by Pearson's correlation analysis. The anatomic structural remodeling of the pharyngeal airway was measured using MRI, including the lager retropalatal distance (*P* = 0.018) and shorter length of soft palate (*P* = 0.044) compared with the baseline. Hence, oropharyngeal muscle exercise is a promising alternative treatment strategy for stroke patients with moderate OSAS.

**Clinical Trial Registration:** http://www.chictr.org.cn. Unique identifier: ChiCTR-IPR-16009970

## Introduction

Based on previous studies, 57% of stroke patients suffer from obstructive sleep apnea syndrome (OSAS) in rehabilitation units (1, 2). OSAS is associated with lower cognitive ability (3), motor ability (4), and daily activity (5) in patients admitted for stroke rehabilitation. Early use of continuous positive airway pressure (CPAP) appears to accelerate neurological recovery and delay cardiovascular events in patients with ischemic stroke (6). However, multiple randomized trials have demonstrated that the efficacy of CPAP has been limited due to poor clinical acceptance and adherence (7, 8). The poor treatment adherence, 12–15% as previously reported (9, 10), is a major limitation resulting in the overall poor efficacy for CPAP. Therefore, finding alternative therapies should help alleviate obstruction in the upper airway and improve compliance. Multiple imaging modalities have been used to study the airway passage and have demonstrated anatomical differences between patients with and without OSAS for physiologic dysfunction of muscles (10, 11). Electromyographic findings suggest reduced pharyngeal muscles and lingualis motility in patients with ischemic stroke (12, 13). Recent studies have demonstrated that training the upper airway muscles can ameliorate moderate OSAS (14, 15). The set of oropharyngeal exercises used in the present study was developed in 16 years and has previously been shown to be effective in patients with stroke-free OSAS in uncontrolled and controlled studies (14–16). However, its therapeutic effect on post-stroke OSAS has not been studied. The present study focused on patients with OSAS in stroke rehabilitation units. The primary aim of the study was to assess the effects of oropharingeal muscle exercises in obstruction severity. The secondary aims were to evaluate the effects of the exercises on recovery of neurological function, sleeping, and anatomic changing of upper airway. It was hypothesized that oropharyngeal muscle exercise could be beneficial for patients' recovery in the airway obstruction and in the motor, neurocognitive, and ADL performance after stroke.

## Methods

### Trial design and oversight

The present study was an open-label, parallel-group, randomized, controlled trial (Clinical Trial Registration; http://www.chictr.org.cn. Unique identifier: ChiCTR-IPR-16009970). It was approved by the ethics committee of the affiliated Zhongshan Hospital of Dalian University in February 2016 (approval number 2016040). Patients were recruited from November 2016 to November 2017. All participants provided informed written consent.

### Inclusion and exclusion criteria

**Figure 1:**
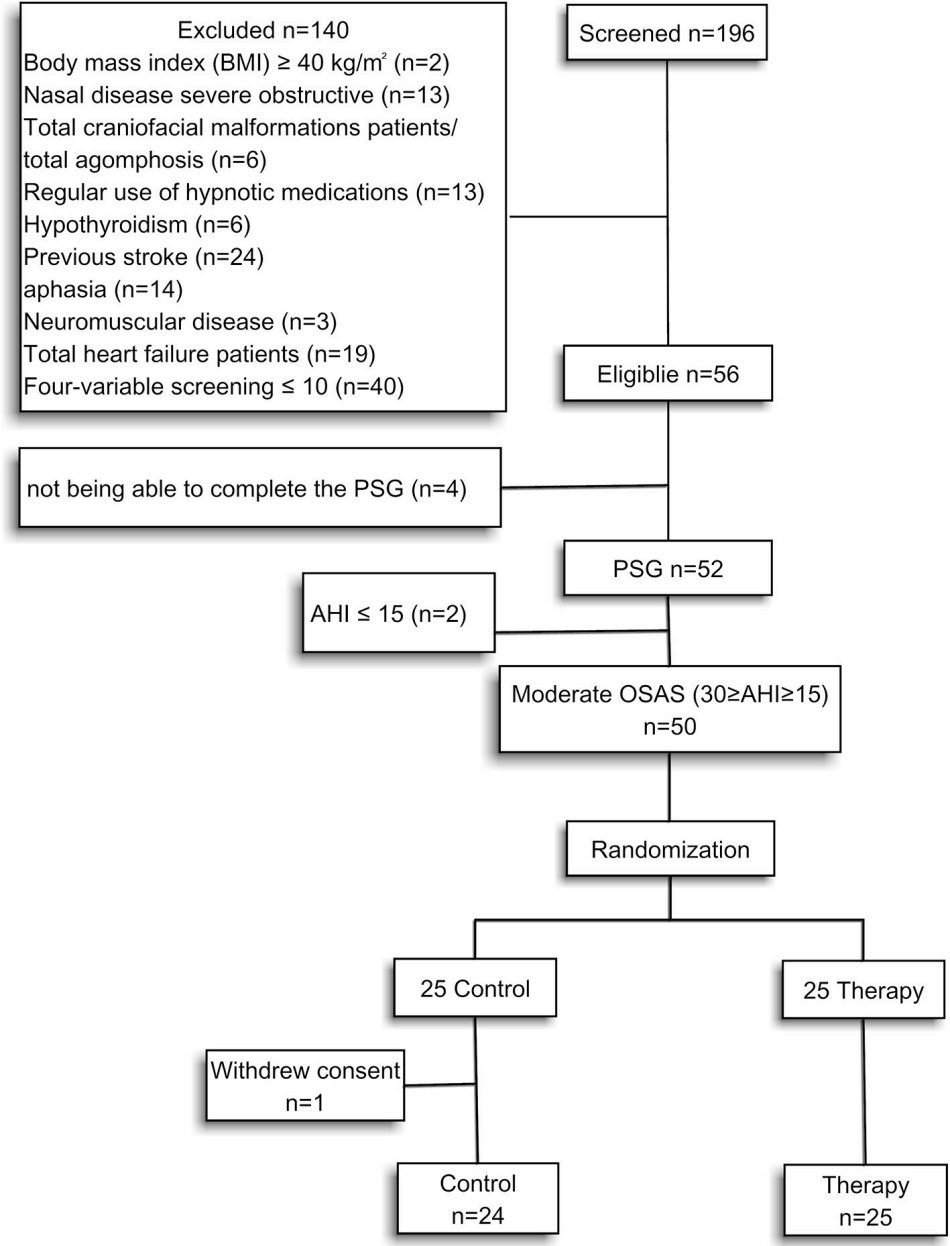
Research design.

Stroke patients visiting the rehabilitation center of the affiliated Zhongshan Hospital of Dalian University were recruited for the study from November 2016 to November 2017. Patients who experienced a first stroke that was either hemorrhagic or ischemic within 3 weeks were scheduled for stroke rehabilitation. The inclusion criteria included patients who snored, had the ability to participate in the sleep study, and complete the functional assessment. The patients' AHI should be >15 and <30 events/hour by polysomnography (PSG). Patients were excluded based on one or more of the following criteria: body mass index (BMI) 40 kg/m^2^ or greater, craniofacial malformations, agomphosis, severe nasal disease, regular use of hypnotic medications, hypothyroidism, previous stroke, aphasia, neuromuscular disease, heart failure, or severe obstructive nasal disease. Sleep-disordered breathing was evaluated using a simple four-variable screening tool. The patients with screening scores below 10 were excluded (17, 18) (Figure 1).

### Sleep apnea diagnosis

A respiratory sleep study was performed in the hospital ward within the first 48–72 h after transferring from the stroke unit to the rehabilitation unit. PSG was performed by trained staff at the patient's bedside before and after therapy. The PSG included electroencephalogram, respiratory inductive plethysmography, electrocardiogram, and oxygen saturation (SaO_2_%). Portable unattended PSG (LifeShirt, Vivometrics, CA, USA) was used for the measurements (1). Apnea was defined as the reduction in peak signal excursion by ≥90% of the pre-event baseline that lasted for ≥10 s. Hypopnea was defined as a reduction in the peak signal excursion of ≥30% for ≥10 s with a corresponding oxygen desaturation event of ≥4%. The AHI was calculated using the total apneic and hypopneic events detected per hour of the recorded night data. Sleep apnea was diagnosed when the AHI ≥5 events/hour. Respiratory events in the presence of abdominal effort were scored as obstructive, and respiratory events in the absence of abdominal effort were scored as central events. A respiratory event was scored as a mixed apnea if it met the apnea criteria and was associated with the absence of inspiratory effort in the second portion of the event. For the purpose of distinguishing between obstructive and central sleep apnea, mixed apneas were classified as obstructive respiratory events (1).

### Intervention

Oropharyngeal muscle exercises were derived from speech–language pathology and included soft palate, tongue, and facial muscle exercises. Patients were instructed by a single speech pathologist to perform the following tasks (14). The concrete proposal of the exercises see the Table 1. Patients would receive assistance from the therapist if they were unable to complete the motion or performed it inadequately. All the exercises were included in a 20-min training session and administered twice a day to the patients in the therapy group. The control group was subjected to 20-min deep breathing training twice a day.

**Table 1:** Oropharyngeal muscle exercises record.

**Motion requirement**	**Times or duration**	**completion status**
(1) pronouncing an oral vowel intermittently and continuously;	5 times	Yes/No
(2) brushing the superior and lateral surfaces of the teeth by tongue;	5 times	Yes/No
(3) placing the tip of the tongue against the front of the palate and sliding the tongue backward;	2 min	Yes/No
(4) forced tongue sucking upward against the palate, pressing the entire tongue against the palate;	2 min	Yes/No
(5) forcing the back of the tongue against the floor of the mouth while keeping the tip of the tongue in contact with the inferior incisive teeth;	2 min	Yes/No
(6) extended tongue;	200 times	Yes/No
(7) orbicularis oris muscle pressure with the mouth closed;	30 s × 4	Yes/No
(8) gargle without water;	200 times	Yes/No
(9) suction movements contracting only the buccinator;	5 s × 10	Yes/No
(10) ice stimulation on the soft palate, palatal arch, tongue root, and posterior wall of the pharynx.	5 s × 10	Yes/No

### Randomization

All the patients were randomized to receive either conventional treatment for stroke combined with oropharyngeal muscle exercise (therapy group) or deep breathing (control group) using a computer-generated random list (1:1 ratio) for 6 weeks. Patients' training time was recorded by the speech pathologist.

### Outcomes

The primary outcomes were obstruction severity by PSG including apnea–hypopnea index (AHI), minimum SaO_2_%, arousal index per sleep hour (AI), and snore index (SI). Several domains were evaluated as secondary outcomes. Motor recovery was evaluated using the Fugl–Meyer assessment (FMA), which included the upper or lower extremity motor performance (19). ADL was assessed by one occupational therapist using the basic Barthel Index (20). Cognition recovery was assessed using the Mini-Mental State Examination (MMSE) scale (21). Secondary outcomes also included sleep quality, sleepiness, and airway MRI measurements. The Stanford Sleepiness Scale (SSS) was administered to assess the level of sleepiness (22). The Pittsburgh Sleep Quality Index (PSQI) was used to determine the participants' sleep habits and quality (23). The participants with OSAS completed the functional, motor, and sleeping quality assessments between 9:00 and 11:00 a.m. administered by individuals who were blinded to subject randomization. Assessment scales were completed before and at the end of the intervention. Assessments were made at baseline and after 6-week exercise.

### Airway MRI measurements

**Figure 2:**
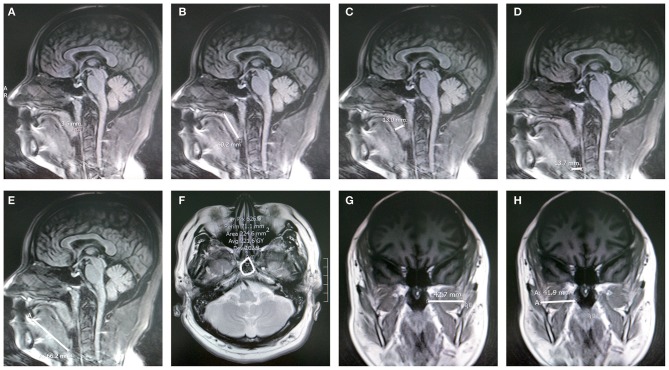
MRI measurements. **(A)** Retropalatal distance on a sagittal T1-weighted image. **(B)** Palatal length on a sagittal T1-weighted image. **(C)** Palatal thickness on a sagittal T1-weighted image. **(D)** Retroglossal space on a sagittal T1-weighted image. **(E)** Tongue length on a sagittal T1-weighted image. **(F)** Cross-sectional area of the retropharyngeal region on an axial T2-weighted image. **(G,H)** Lateral pharyngeal wall thickness on a coronal T1-weighted image.

The brain MRI examination was performed on 30 patients (15 from each group) at the beginning and end of the intervention. Images were obtained in the supine position with the head in the neutral position. Brain MRI scans were retrieved from the hospital's PACS archives, and measurements were performed using the syngo.via image software (Siemens). Experienced neuroradiologists who were blinded to the interventions performed all the measurements. Measurements included the retropalatal distance (midsagittal T1), soft palatal length (midsagittal T1), soft palatal thickness (midsagittal T1), retroglossal space (midsagittal T1), tongue length (midsagittal T1), nasopharyngeal area (axial T2), and lateral pharyngeal wall thickness (coronal T1) (24) (Figure 2).

### Statistical analysis

At least 17 patients in each group were required to determine a 50% reduction in objective snoring in patients randomized to oropharyngeal exercises based on a previous study, using a two-sample design and the assumption of an α of 0.05 and 80% power (14). Data were expressed as mean [standard deviation (SD)] for normal distribution and as median (interquartile range 25–75%) for skewed variables. Data were analyzed using SPSS (version 22.0, IBM, NY, USA). The variables were tested for normal distribution using the Kolmogorov–Smirnov test. Variables not normally distributed were logarithmically transformed. Two-factor analysis of variance with adjustment for gender and calendar age was used to compare differences between groups for variables measured at baseline and after 6 weeks. Paired-samples *t*-tests were used to compare the difference in the value within a group between the baseline and after 6 weeks. Effect size values were calculated using Cohen's *d*. The effect size was computed by finding the difference between the control and treatment groups in the outcome measure after 6-week exercises and dividing this value by the pooled SD. As set forth by Cohen (1988), an effect size <0.2 was considered a small effect; 0.21–0.5 was a medium effect; and >0.8 was a large effect. The changes in PSG parameters, sleeping characteristics, performance scale, and MRI measurements were calculated using the difference in the values between 6 weeks and onset. The association between the changes and training time was assessed using the Pearson's correlation analysis. The correlation coefficient <0.2 was considered an extremely weak correlation or no correlation, 0.21–0.4 was considered a weak correlation, 0.41–0.6 was considered a moderate correlation, 0.61–0.8 was considered a strong correlation, and 0.41–0.6 was considered an extremely strong correlation (25). A *P* < 0.05 was considered significant.

## Results

A total of 196 stroke patients with snoring complaints were screened in this study. Fifty-six patients with screening scores above 10 were administered a polysomnography (PSG). Four patients who were unable to complete the PSG were excluded. Only two patients had an AHI ≤ 15. They were excluded for maintaining consistency of the moderate of apnea. Finally, 50 patients were selected for the study with 30 ≥ AHI ≥ 15 events/hour. One patient in the control group withdrew due to recurrent strokes (Figure 1). The remaining patients achieved 81.5–100% of the expected training time. The demographic, sleep characteristics and training time, according to the group assigned, are presented in Table 2. Patients assigned to the control and therapy groups had similar baseline characteristics (Table 2). Obstructive sleep apnea was the predominant respiratory event, with central apneas being rare, while none of the patients had pure central sleep apnea.

**Table 2:** Anthropometric data and baseline characteristics of the total sample.

**Characteristic**	**Control (*n* = 24)**	**Therapy (*n* = 25)**	***P*-value**
Age	65.5 (10.4)	63.4 (9.9)	0.449^a^
Male, no.	17 (70.8)	19 (76.0)	0.399^b^
BMI, kg/m^2^	27.8 (3.2)	26.7 (2.8)	0.182^a^
Neck circumference, cm	43.0 (42.0, 45.0)	43.0 (42.0, 44.5)	0.120^c^
NIHSS	8.4 (2.7)	7.7 (2.6)	0.370 ^a^
**MEDICAL HISTORY**			
Snore history	18 (75.0)	16 (64.0)	0.404^b^
Hypertension	21 (87.5)	25 (100)	0.068^b^
Diabetes	14 (58.3)	13 (52.0)	0.656^b^
Hyperlipidemia	13 (54.2)	16 (64.0)	0.484^b^
Atrial fibrillation	7 (29.2)	11 (44.0)	0.282^b^
Current smoker	17 (70.8)	14 (56.0)	0.291^b^
Stroke type			0.102^b^
ischemia	21 (87.5)	17 (68.0)
Hemorrhage	3 (12.5)	8 (32.0)
**PERFORMANCE SCALE**			
MMSE	24.8 (2.4)	25.8 (2.5)	0.172^a^
FMA	33.3 (8.2)	30.5 (9.2)	0.266^a^
Barthel Index	30.0 (27.5, 37.5)	30.0 (26.6, 35.0)	0.837^c^
**SLEEP CHARACTERISTICS**			
SSS	6.0 (5.0, 6.0)	6.0 (5.0, 6.0)	0.675^c^
PSQI	8.0 (1.7)	7.6 (1.8)	0.750^a^
**PSG RESULTS**			
AHI	20.1 (3.0)	20.8 (4.0)	0.494^a^
Minimum SaO_2_, %	81.2 (3.3)	80.8 (4.6)	0.724^a^
AI	17.3 (3.2)	18.2 (3.8)	0.411^a^
SI	19.4 (4.4)	18.4 (4.4)	0.472^a^
Central facial paralysis	12 (50.0)	18 (72.0)	0.114^b^
Days between onset to rehab unit	11.9 (2.6)	12.3 (3.5)	0.656 ^a^
Training time, min	1482.7 (108.0)	1513.2 (102.7)	0.306^a^

*BMI, body mass index; NIHSS, NIH Stroke Scale; MMSE, Mini-mental State Examination; FMA, Fugl-Meyer Assessment; SSS, Stanford sleepiness scale; PSQI, Pittsburg sleep quality index scale; PSG, polysomnogram; AHI, apnea-hypopnea index; AI, arousal index per sleep hour; SI, snore index. Values are presented as mean (standard deviation), n (%) or median (interquartile range)*.

a *Student t-test*.

b *Fisher exact test*,

c *Mann-Whitney U test*.

### Primary outcomes

After 6 weeks of treatment, no significant changes were observed in the control group; however, significant changes were observed in the therapy group (Table 3). In contrast, patients who were randomized into the oropharyngeal muscle exercise group had a significant decrease in AHI (*P* = 0.004), AI (*P* = 0.010), and SI (*P* = 0.006), and an increase in minimum SaO_2_% (*P* = 0.039) between the control and therapy groups with adjustment for gender and calendar age (Table 3). The values of effect size for AHI, AI, SI, and minimum SaO_2_% were all > 0.8, indicating a large effect of exercises on the improvement in airway obstruction (Table 4). After the Pearson's correlation analysis, moderate correlations were found between the training time of oropharyngeal exercises and the changes in SI, AI, minimum SaO_2_% (all *P* < 0.05), and a strong correlation between the training time and AHI change (*r* = −0.833, *P* < 0.001) (Table 5).

**Table 3:** Polysomnographic, sleeping, and performance data.

	**Control**		**Therapy**		***P*-value**
	**Baseline**	**6 weeks**	**Baseline**	**6 weeks**
AHI	20.1 (3.0)	19.5 (3.5)	20.8 (4.0)	14.3 (4.1)†#	0.004
Minimum SaO_2_, %	81.2 (3.3)	82.2 (4.2)	80.8 (4.6)	85.8 (4.2)†#	0.039
AI	17.3 (3.2)	16.9 (3.8)	18.2 (3.8)	13.0 (3.3)†#	0.010
SI	19.4 (4.4)	18.2 (4.5)	18.4 (4.4)	13.7 (5.6)†#	0.006
SSS	6.0 (5.0, 6.0)	5.5 (5.0, 6.0)	6.0 (5.0, 6.0)	4.0 (3.0, 4.5)†#	0.005
PSQI	8.0 (1.7)	7.6 (1.8)	7.6 (1.8)	5.7 (1.9)†#	0.017
MMSE	24.8 (2.5)	26.1 (1.9)†	25.8 (2.6)	26.9 (2.2)†	0.741
FMA	33.3 (8.2)	42.0 (10.3)†	30.5 (9.2)	57.5 (15.2)†#	0.006
Barthel Index	30.0 (27.5, 37.5)	45.0 (35.0, 55.0)†	30.0 (26.6, 35.0)	60.0 (51.3, 70.0)†#	<0.001

*AHI, apnea-hypopnea index; SaO_2_, oxygen saturation; AI, arousal index per sleep hour; SI, snore index; SSS, Stanford sleepiness scale; PSQI, Pittsburg sleep quality index scale; MMSE, Mini-mental State Examination; FMA, Fugl-Meyer Assessment. Values are presented as mean (standard deviation) or median (interquartile range)*.

† *P < 0.05 baseline vs. 6 weeks*.

# *P < 0.05 control (6 weeks) vs. therapy (6 weeks)*.

**Table 4:** The effect size measure for the difference between the groups after 6 weeks exercise.

	Control^*^	Therapy^*^	***P*-value**	***t*-value**	**Cohen's *d* (95% confidence intervals)**
AHI	19.5 (3.5)	14.3 (4.1)	0.000	4.615	1.319 (0.693, 1.933)
Min SaO2, %	82.2 (4.2)	85.8 (4.2)	0.001	−3.520	1.006 (0.406, 1.597)
AI	16.9 (3.8)	13.0 (3.3)	0.003	3.146	0.899 (0.306, 1.483)
SI	18.2 (4.5)	13.7 (5.6)	0.000	4.366	1.248 (0.628, 1.856)
SSS	5.5 (5.0, 6.0)	4.0 (3.0, 4.5)	0.001	3.503	1.001 (0.401, 1.592)
PSQI	7.6 (1.8)	5.7 (1.9)	0.000	−1.216	1.187 (0.573, 1.791)
MMSE	26.1 (1.9)	26.9 (2.2)	0.230	−4.155	0.348 (−0.219, 0.910)
FMA	42.0 (10.3)	57.5 (15.2)	0.000	−4.941	1.415 (0.780, 2.037)
Barthel index	45.0 (35.0, 55.0)	60.0 (51.3, 70.0)	0.000	4.615	1.319 (0.692, 1.933)

*AHI, apnea-hypopnea index; SaO_2_, oxygen saturation; AI, arousal index per sleep hour; SI, snore index; SSS, Stanford Sleepiness Scale; PSQI, Pittsburg sleep quality index scale; MMSE, Mini-mental State Examination; FMA, Fugl-Meyer Assessment*,

* *Values are presented as mean (standard deviation) or median (interquartile range)*.

**Table 5:** Correlation of the training time and the change of PSG parameter, sleep characteristics, and performance in Pearson's correlation analysis.

	**The length of training time**	
	***r***	***P*-value**
Δ AHI	−0.833	<0.001
Δ Min SaO_2_	0.487	0.014
Δ AI	−0.541	0.035
Δ SI	−0.605	0.001
Δ SSS	−0.450	0.024
Δ PSQI	−0.462	0.020
Δ MMSE	0.358	0.079
Δ FMA	0.627	0.001
Δ Barthel index	0.569	0.003

*Δ, 6 weeks – baseline; AHI, apnea – hypopnea index; SaO_2_, oxygen saturation; AI, arousal index per sleep hour; SI, snore index; SSS, Stanford sleepiness scale; PSQI, Pittsburg sleep quality index scale; MMSE, Mini-mental State Examination; FMA, Fugl-Meyer Assessment*.

### Secondary outcomes

Patients assigned to the exercise group experienced a significant increase in motor function (FMA) (*P* = 0.006) and basic ADL score (Barthel Index) (*P* < 0.001) (Table 3), implying a large effect (Table 4). Despite an increase in cognitive function (MMSE), no significant difference between the two groups was observed. The results of Pearson's correlation analysis indicated that there were moderate correlations between the training time of oropharyngeal exercises and the changes in basic ADL scores (*r* = 0.569, *P* = 0.003), and a strong correlation between the training time and FMA change (*r* = 0.627, *P* = 0.001). However, no correlation was found between the training time and MMSE (*r* = 0.358, *P* = 0.079) (Table 5). The exercises significantly improved the scores for the sleep quality questionnaires (*P* = 0.017). The results of SSS decreased (*P* = 0.005), indicating that the excessive daytime sleepiness reduced (Table 3). The values of effect size for SSS and PSQI were >0.8, implying a large effect of exercises on the improvement in sleeping quality and daytime sleepiness (Table 4). Comparisons of MRI measurements between the therapy and control groups are shown in Table 6. After therapy, the retropalatal distance was significantly larger in the therapy group than in the control group (*P* = 0.018). The length of the soft palate was also found to be reduced significantly in the therapy group than in the control group (*P* = 0.044). Regarding the difference in the outcome measure after 6-week exercises between the control and treatment groups, the values of effect size showed that the exercises had a large effect on the retropalatal distance and soft palate length and small effects on other measurements (Table 7). The results of the Pearson's correlation analysis demonstrated the association between the MRI measurement changes and the training time of oropharyngeal exercises (Table 8). Larger retropalatal distance (*r* = 0.800, *P* < 0.001) and shorter soft palates (*r* = −0.747, *P* = 0.001) were strongly associated with longer training times.

**Table 6:** MRI measurement data (mm or mm^2^).

	**Control**		**Therapy**		***P*-value**
	**Baseline**	**6 weeks**	**Baseline**	**6 weeks**
RPD	1.7 (0.1)	1.8 (0.2)	1.7 (0.1)	2.1 (0.2)†#	0.018
SPL	50.2 (3.1)	50.3 (4.8)	49.2 (2.7)	45.5 (1.9†#	0.044
MPT	9.9 (1.4)	10.0 (1.5)	9.6 (1.8)	10.0 (1.3)	0.656
RGS	9.1 (1.7)	10.0 (1.8)†	9.5 (2.2)	10.1 (1.8)†	0.747
TL	67.1 (8.7)	67.6 (6.8)	66.1 (8.4)	66.2 (9.4)	0.951
NPA	351.9 (24.5)	355.2 (22.6)	355.6 (28.2)	362.6 (32.5)	0.781
LPWT	79.4 (7.3)	78.9 (6.6)	79.2 (8.0)	80.3 (7.5)	0.659

*RPD, Retropalatal distance; SPL, Soft palate length; MPT, Maximum palatal thickness; RGS, Retroglossal space; TL, Tongue length; NPA, Nasopharyngeal airway (mm^2^); LPWT, lateral pharyngeal wall thickness*.

† *P < 0.05 baseline vs. 6 weeks*.

# *P < 0.05 control (6 weeks) vs. therapy (6 weeks)*.

**Table 7:** The effect size measure for the difference between the groups after 6 weeks exercise.

	Control^*^	Therapy^*^	***P*-value**	***t*-value**	**Cohen's *d* (95% confidence intervals)**
DPR	1.8 (0.2)	2.1 (0.2)	0.000	4.02	1.468 (0.646, 2.269)
SPL	50.3 (4.8)	45.5 (1.9)	0.002	3.33	1.214 (0.423, 1.988)
MPT	10.0 (1.5)	10.0 (1.3)	0.886	−0.145	0.053 (−0.664, 0.769)
RGS	10.0 (1.8)	10.1 (1.8)	0.846	−0.195	0.071 (−0.645, 0.787)
TL	67.6 (6.8)	66.2 (9.4)	0.613	0.512	0.187 (−0.532, 0.903)
NPA	355.2 (22.6)	362.6 (32.5)	0.462	−0.745	0.272 (−0.450, 0.989)
LPWT	78.9 (6.6)	80.3 (7.5)	0.571	−0.573	0.209 (−0.510, 0.925)

*RPD, Retropalatal distance; SPL, Soft palate length; MPT, Maximum palatal thickness; RGS, Retroglossal space; TL, Tongue length; NPA, Nasopharyngeal airway (mm^2^); LPWT, lateral pharyngeal wall thickness*.

* *Values are presented as mean (standard deviation)*.

**Table 8:** Correlation of the training time and MRI measures change in Pearson's correlation analysis.

	**The length of training time**	
	***r***	***P*-value**
Δ RPD	0.800	<0.001
Δ SPL	−0.747	0.001
Δ MPT	0.200	0.474
Δ RGS	−0.043	0.879
Δ TL	−0.071	0.801
Δ NPA	0.067	0.814
Δ LPWT	0.333	0.225

*Δ, 6 weeks-baseline; RPD, Retropalatal distance; SPL, Soft palate length; MPT, Maximum palatal thickness; RGS, Retroglossal space; TL, Tongue length; NPA, Nasopharyngeal airway (mm^2^); TLPT, Total lateral pharyngeal soft tissue thickness*.

## Discussion

The present study examined the effects of oropharyngeal exercise in stroke patients with moderate OSAS using objective polysomnography measurements and quantification of subjective symptoms, including snoring, daytime sleepiness, and sleep quality. Good compliance was observed in participation and time spent on the exercises. A satisfactory therapeutic effect of oropharyngeal exercise on OSAS was demonstrated by the decrease in AHI, AI, and SI and increase in minimum SaO_2_%, suggesting the improvement in upper airway obstruction. According to the PSG results (14 ≥ AHI ≥ 5, 89 ≥ SaO_2_% ≥ 85) (26), 52.0% (13 in 25) of subjects in treatment group was diagnosed with mild OSAS comparing with 4.1% (1 in 24) in control group after 6 weeks exercises. In cases of severe and moderate OSAS, treatment is necessary and CPAP is the first-line option (27). Significant increasing were observed on oxygen saturation CPAP titration (more than 15%), and also 90% decrease was recorded on AHI in patients with stroke-free OSAS (28). However, CPAP treats OSAS but is generally poorly tolerated by stroke patients. The result of previous study on nasal expiratory positive airway pressure (EPAP) for sleep apnea after stroke shew a 33.5% decrease on AHI and a 11.4% increase on minimum SaO_2_% (29). The results of the present research (a 25.5% decrease on AHI and a 6.19% increase on minimum SaO_2_%) indicate that the effect of oropharyngeal exercise on improvement of airway obstruction seems to lower than CPAP and EPAP.

The genesis of post-stroke OSAS is multifactorial and includes anatomic and physiological factors. Upper airway dilator muscles are crucial for maintaining pharyngeal patency and may contribute to the genesis of OSAS (30). The influence of oropharyngeal exercise on the upper airway anatomy was explored in the present study. Retropalatal distance increased significantly after exercise. Additionally, the soft palate measurements showed that the obstruction in the pharyngeal morphology improved after exercise. The clinical significance of the change in upper airway anatomy was confirmed by effect size (Cohen's *d*). A previous study demonstrated that shorter retropalatal distances were associated with higher AHIs (24). Longer vertical soft palatal dimensions were observed in both non-stroke and stroke patients with OSAS (11, 24). The larger retropalatal distances and shorter soft palate length related to tonic activity were thought to enhance the stiffness in the rostral upper airway. Hence, it was believed that obstruction in the upper airway was alleviated by the exercises via the increase in muscular tension of the pharyngeal muscles. Epidemiological studies on stroke patients have shown that hypoactivation of the tongue is the major mechanism underlying narrowing and closing of the upper airway (14, 31). The neural modulation and motor output to the genioglossus muscle of the tongue are weak in wakefulness and natural sleep (32). Anatomically, movement of the tongue not only engages the lingualis but also the pharyngeal muscles around it. In the present study, 5 of the 10 movement exercises were related to the lingualis. Although the length of the tongue did not change significantly on MRI, the lingualis and pharyngeal muscles improved after exercise. A recent study indicated that single tongue treatment by intraoral electrical neurostimulation could reduce snoring but not AHI (33). In the present study, oropharyngeal and lingual muscle exercises reduced AHI. Hence, the muscle groups exercised for the upper airway benefited stroke patients with OSAS.

The training time of oropharyngeal muscle exercise was an important factor, as a kind of kinetotherapy. Both therapeutic effects and a good compliance need to be taken into account. Ieto et al. instructed patients to perform oropharyngeal exercises three times a day, including the six mastication patterns for approximately 8 min. Oropharyngeal exercises were effective in reducing objectively measured snoring (15). Guimarães's 30-min oropharyngeal exercises included six actions for linguales, five for facial muscles, and one for swallowing and chewing. It improved the snoring frequency, daytime sleepiness, and neck circumference (14). In Puhan's study on didgeridoo-playing treatment for OSAS, participants had to practice at home for at least 20 min on at least 5 days a week, which improved the daytime sleepiness and AHI (34). In the present study, the training time was 20 min twice a day. All the patients attainted 81.5–100% of training time. A positive correlation existed between the improvement in performance and training time. According to the results of Pearson's correlation analysis, reextension of time or frequency might lead to further improvement. However, the compliance likely declined because the exercise was a bit tedious in spots.

Previous studies demonstrated that OSAS was an independent predictor for worse functional outcome in post-stroke patients (35, 36). In a previous study, respiratory sleep disorders correlated with early neurological deterioration and less satisfactory functional outcomes as evaluated using the Barthel Index (37). Patients with recent stroke and sleep apnea were found to have a poorer functional outcome assessed using the Barthel Index at discharge from rehabilitation and 3 and 12 months after stroke onset (38). The present study focused on whether effective treatment of OSAS after stroke improved functional outcome. The results showed that recovery of motor function improved after oropharyngeal muscle exercise. The physical ADL estimated using the Barthel Index also improved significantly after the exercise intervention. Previous randomized studies on subacute stroke patients (in the range of 2–4 weeks) demonstrated the beneficial effects of CPAP on sleep architecture, motor function, and ADL (4, 7). The functional independence and motor improvements might have alleviated the adverse cerebrovascular effects of OSAS, possibly through enhanced neuroplasticity. A previous study found that unilateral nasal obstruction in rats during growth periods induced changes in arterial SaO_2_% and altered the development of motor representation within the face primary cortex (39). The greater reduction in alveolar ventilation during REM sleep also resulted in transient declines in SaO_2_% from a sustained level of hypoxia during NREM sleep for OSAS. Patients with OSAS demonstrated increased motor cortex inhibition by increasing GABAergic tone (40). The results of PSG in both previous studies and the present study indicated that the apnea–hypopnea events and minimum SaO_2_% were ameliorated by both CPAP and oropharyngeal exercise. Therefore, the promotion of motor function and functional independence might enhance neuroplasticity by oropharyngeal muscle exercise, which may result from its positive cerebral SaO_2_% effects. However, the difference in cognitive function after exercise between the two groups was not significant, which was also observed in studies using CPAP therapy for stroke patients with OSAS (7, 36, 41). The exact mechanism has yet to be deciphered and needs more investigation.

The present study had some limitations. First, patients enrolled in the study were required to complete the questionnaire evaluation and oropharyngeal muscle exercises. Patients with significant physical impairment or aphasia were excluded. Thus, the results of this study may not be applicable to patients with severe strokes. Second, based on the inclusion and exclusion criteria, most of the patients enrolled had moderate OSAS (AHI from 15 to 30). The therapeutic efficacy of exercise in mild and severe OSAS should be investigated further. Third, the sleep status of patients during MRI was not documented. Differences in MRI measurements on airway have been observed in awake patients with and without OSAS (24). Hence, it is believed that this method can be used to evaluate changes in the anatomic structures of the airway. Finally, CPAP is the clinically recommended treatment for stroke patients with OSAS. Unfortunately, the therapeutic efficacy of exercise and CPAP could not be compared due to limited resources. This is an important comparison and should be conducted in future studies.

## Conclusions

In conclusion, oropharyngeal muscle exercises reduced OSAS severity and increased sleep quality in stroke patients with moderate OSAS. The oropharyngeal exercise also improved the rehabilitation of motor function and personal ADL. The anatomic structural remodeling of the upper airway (larger retropalatal distance and shorter soft palate) observed by MRI improved after exercise. The results suggested that the set of oropharyngeal muscle exercises used in the present study may serve as a promising alternative therapeutic strategy for stroke patients with moderate OSAS.

## Author contributions

DY and MS conceived and designed the study. DS, SZ, HZ, JL, and WY performed the experiments. CC, HL, and QW analyzed the data. DY and CC wrote the manuscript.

### Conflict of interest statement

The authors declare that the research was conducted in the absence of any commercial or financial relationships that could be construed as a potential conflict of interest. The reviewer MG and handling Editor declared their shared affiliation at the time of the review.
